# Infrapatellar Saphenous Nerve Is at Risk During Tibial Nailing: An Anatomic Study

**DOI:** 10.5435/JAAOSGlobal-D-21-00007

**Published:** 2021-10-04

**Authors:** Glenn G. Shi, Arun R. Kumar, Cameron K. Ledford, Cedric J. Ortiguera, Benjamin K. Wilke

**Affiliations:** From the Department of Orthopedic Surgery, Mayo Clinic, Jacksonville, FL.

## Abstract

To determine the location and stage of the tibial nailing procedure where infrapatellar saphenous nerve (IPSN) injury may occur.

**Methods:**

Fourteen fresh-frozen right cadaveric knees underwent tibial nailing. Six knees underwent a suprapatellar approach and 8 a medial parapatellar approach. Two proximal medial-to-lateral screws were placed using the aiming guide. The incisions were then closed. After the procedure, medial retinacular and saphenous nerves were dissected under surgical 2.5× loupe magnification from a proximal to distal direction. The branch of the IPSN closest to the locking screws was measured, as was the distance between the IPSN branch and the inferior pole of the patella.

**Results:**

Twelve of 14 cadavers had prominent IPSN (main branch from the saphenous proper) with an average of 2.5 sub-branches. The mean (SD) distance from the main branch of the IPSN to the inferior pole of the patella was 40.9 (24.4) mm. Four medial retinacular nerve branches, branching from the femoral nerve and not IPSN, were identified proximal to the patella during the medial parapatellar approach. All were cut after the medial parapatellar arthrotomy. The mean (SD) distance from the IPSN to the nearest locking screw was 10.2 (14.1) mm. Seven of 14 had IPSN injuries, and one had hamstring injury. Two direct screw entrapments occurred, whereas two IPSNs were lacerated by the incision. Suture closure entrapped three nerve branches, and one specimen had injured fibers of the hamstring tendinous insertion.

**Conclusions:**

Injury to the IPSN can occur at different locations and stages of tibial nailing, including approach, proximal locking screw insertion, and closure.

Intramedullary nail is an accepted treatment for tibial shaft fractures with reliable union. A common postsurgical result, more commonly associated with infrapatellar approach, is anterior knee pain after the placement of the intramedullary nail, occurring in up to 47.4% of patients.^1^ This pain has often been theorized to be from prominent implant, cartilage injury, fat pad damage, or fibrosis^2^; however, anterior knee pain may also be caused by infrapatellar saphenous nerve (IPSN) injury.^3^ The infrapatellar branch of the saphenous nerve originates out of the adductor canal in the medial distal thigh. Through various patterns that have been previously described through anatomic studies, we understand that the distribution is highly variable, although much of the anterior knee is supplied by branches of the IPSN.^4^ Incisions for surgical implant placement through this area can create an opportunity for iatrogenic nerve injury, resulting in hypersensitive neuropathic pain. Previous studies suggest that injury to the IPSN after tibial nail is substantial and permanent. However, evidence is lacking on when and where the nerve injury occurs during the tibial nailing procedure. The purpose of this anatomic study was to determine the location and stage of the tibial nailing procedure where IPSN injury may occur.

## Methods

Fourteen fresh-frozen right cadaveric knees underwent unreamed tibial nailing (Expert Tibial Nail, 12 mm × 325 mm, DePuy Synthes Companies). The specimens were evaluated before the procedure for any evidence of congenital deformity, previous surgeries, previous injuries, or any other evidence of altered anatomy. Six consecutive knees were assigned to a suprapatellar approach, and eight to a medial parapatellar approach. The suprapatellar approach was based on the Synthes Expert Tibial Nail system, with incision proximal to the patella through the quadriceps tendon with a towel bump under the knee to facilitate exposure. The nail was inserted such that the proximal end of the nail is 0 to 10 mm past the cortex using the proximal aiming arm as a gross guide. The medial parapatellar approach was used to insert the Synthes Expert Tibial Nail to represent the infrapatellar nail insertion method. After skin incision, the medial parapatellar capsulotomy was made along the medial border of the patella to visualize the starting point directly. Two proximal medial-to-lateral locking screws were placed into the intramedullary nail using the aiming guide. The incisions were then closed with sutures in layers. After the procedure, the medial retinacular and saphenous nerves were dissected under 2.5× loupe magnification along their course from a proximal to distal direction. The branch of the IPSN closest to the screws was measured, as was the distance between the IPSN branch and the inferior pole of the patella.

## Results

**Figure 1:**
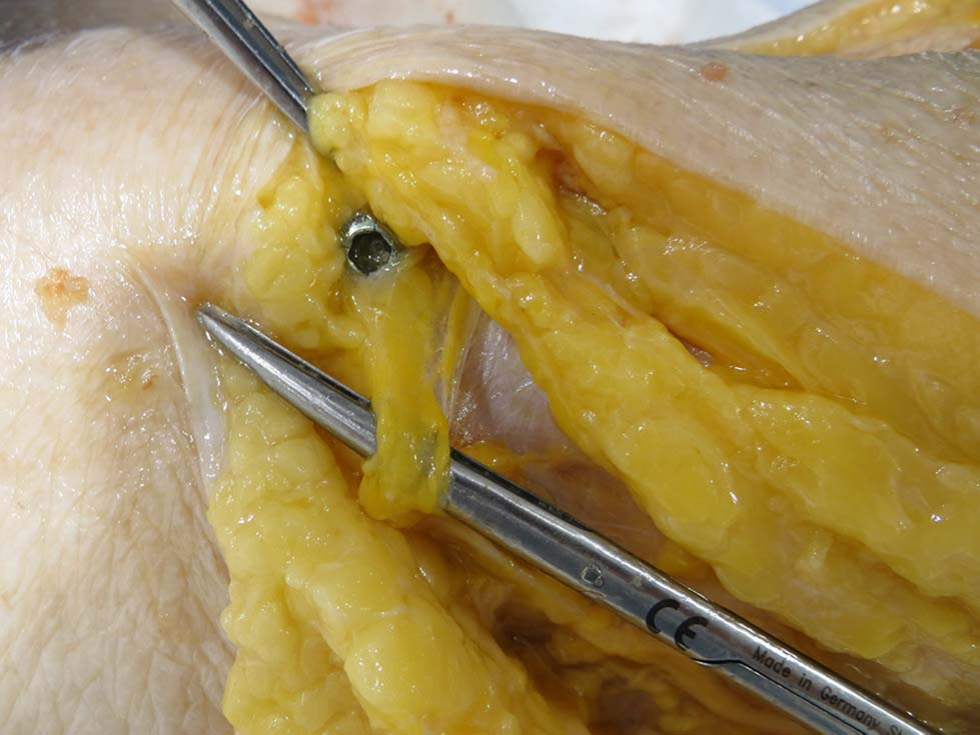
Photograph showing an example of a nerve entrapment in specimen #6 with direct injury by proximal locking screw.

Twelve of 14 cadavers had prominent IPSN with an average of 2.5 branches. The mean (SD) distance from the main branch of the IPSN to the inferior pole of the patella was 40.9 (24.4) mm. Four medial retinacular nerve branches from the femoral nerve were identified proximal to the patella during the medial-parapatellar approach. These were not nerves that traced to the saphenous proper. All were cut after the medial parapatellar arthrotomy. The mean (SD) distance from the IPSN to the nearest proximal locking screw was 10.2 (14.1) mm. Seven of 14 had IPSN injuries, with one injury involving the hamstring with direct screw entrapment (Table 1). Two direct screw entrapments occurred (Figure 1), whereas two IPSNs were lacerated by the incision. Suture closure entrapped three nerve branches, and one specimen had injured fibers of the hamstring tendinous insertion.

**Table 1:** Laterality, Site of Injury, and Distance From Anatomic Landmarks

Specimen	Entry	Side	Nerve location inf patella, mm	Distance to closest screw, mm	Notes
1	S	R	−30	23	Distal locking screw entrapped hamstring
2	M	R	61	31	
3	M	R	51	1	
4	M	R	20	4	
5	S	R	59	1	Branch cut during approach
6	S	R	55	0	Direct injury from proximal screw
7	S	R	42	3	
8	S	R	30	2	Entrapment of fascia over nerve from proximal screw
9	S	R	42	25	
10	M	R	41	3	
11	M	R	27	2	
12	M	R	59	0	Direct injury from distal screw
13	M	L	54	5	
14	M	L	62	43	

inf = Distance inferior to the patella, M = Medial parapatellar, S = Suprapatellar

## Discussion

Postoperative anterior knee pain after tibial nail is a known condition that can cause permanent functional loss and continued pain in up to 47.4% of patients.^1^ To our knowledge, this is the first anatomic study to evaluate the relationship of the IPSN to points of potential injury during intramedullary tibial nailing. In our series, the nerve was injured at several locations: the medial parapatellar approach, with direct laceration along the medial retinacular layer; the proximal medial-to-lateral locking screw placement, with either direct screw injury to the nerve or entrapment of the fascia overlying the nerve; direct incision with the scalpel in the infrapatellar region; and entrapment by closure in layers of the infrapatellar incision.

Sensory deficits and anterior knee pain after intramedullary tibial nailing is a concern, given its prevalence in the clinical setting. Several controversial sources of pain have been previously described.^5-7^ With the recent understanding of IPSN pathways, potential iatrogenic injury to this nerve can lead to permanent neuralgia along the anterior and medial aspect of the knee after surgery. This is especially true with knee surgeries involving the anterior approach or anteromedial surgical incisions. Recent recognition of this in the clinical setting has led to surgeon awareness.^8^ Despite early explorations of skin incisions, whether longitudinal or transverse, postoperative anterior knee pain occurs in 38% to 56% of patients.^4^ Current limited literature has evaluated various approaches, including medial parapatellar, transtendinous, lateral parapatellar, and suprapatellar methods and their respective reports of anterior knee pain.^9^ Both lateral and suprapatellar approaches reflect a lower incidence of reported anterior knee pain.^9,10,11,12^ Pathways of iatrogenic injury may involve direct nerve transection, scar entrapment, and suture entrapment during layered closure. All major branches of the IPSN can be seen with 2.5× surgical loupe magnification, whereas some may be seen even without magnification.

The IPSN pathway is highly variable.^4,13^ The nerve branching pattern is largely unpredictable and does not provide a true safe zone. Multiple patterns occur at the level, crossing the patellar tendon, reaching distally. Therefore, whether the approach is a medial retinacular or transtendinous likely mattered little in iatrogenic injury to the nerve in nonsuprapatellar approach. There was one patient described who did not have an identifiable IPSN branch but did have extensive lateral-sided branching and a single medial retinacular branch at the level of the distal pole of the patella. We conclude this to be a variant.

Four of the eight medial retinacular branches were cut during the medial parapatellar approach for tibial nail insertion. This is notable because these nerves have the potential to develop painful neuromas or be entrapped in the suture closure of the joint capsule, as in three of the four nerves identified. None of these nerves would have been injured through the suprapatellar approach. Conversely, no iatrogenic injury occurred to the quadriceps tendon during the medial parapatellar approach compared with the suprapatellar approach, which requires an incision proximally.

It is important to recognize that IPSN injury is not the only source of anterior knee pain after tibial nailing procedure. Other causes include the Hoffa fat pad trauma, patellar tendon injury, prominent nail, meniscus injury, and patellofemoral chondral injury.^14,15,16,17^ Although the true cause of anterior knee pain is controversial and likely multifactorial, injury avoidance of the medial retinacular and IPSN branches may reduce postoperative neuralgia.^1^ Leliveld et al^8^ evaluated the influence of the IPSN's contribution to postoperative knee pain after infrapatellar approach for tibial nailing in their randomized clinical trial of infrapatellar branch nerve block with local anesthetic versus placebo. They reported a considerable reduction of kneeling pain with the local anesthetic group.^8^ Our anatomic study provides insight as to why the IPSN plays a role as a source of pain and why there could be variations in knee pain after suprapatellar and infrapatellar tibial nailing. Differences in individual nerve branching pattern, types of tibial nails used, techniques, and approaches all can contribute to pain, not to mention the characteristics of the trauma and fracture themselves. In our study, we were able to illustrate, even with a smaller sample, that both suprapatellar and infrapatellar approaches injure the IPSN by the placement of proximal medial-to-lateral locking screws. Suprapatellar approach avoids injury to the medial retinacular nerve branches.

Several limitations exist to our study. First, we used a single tibial nail for all specimens. The nail does not reflect sizing to individual patients as we would otherwise measure. This may have a small impact on the proximal screw placement. Second, the nail was placed without the assistance of fluoroscopy to evaluate the depth of the nail. However, we believe this does not affect the nature of the study, given that all nails were visually inspected to ensure the proper depth and rotation of the placement before the insertion of proximal locking screws. Third, the surgeons involved in the nailing procedure and nerve dissection were not blinded. Fourth, we had a relatively small sample size with both suprapatellar and infrapatellar approaches. Fifth, we used one brand of tibial nail available to our institution. Some may argue that these results would not translate to other manufacturers. We do not think this would make a notable change in the current outcomes, given the high variability of the nerve branching pattern, and the similarities of the proximal locking screw positions, with multiple screw slots between 17 and 57 mm from the proximal end of the tibial nail. Last, the translational ability to clinical practice is lacking, given that not all nerve injuries equate to neuralgia or permanent pain.

## Conclusion

Injury to the IPSN can occur at different locations and stages of the procedure, including the medial-parapatellar approach, proximal locking screw insertion, skin incision, and closure. True loss of function after injury to the nerve branch is difficult to measure clinically. Future design features of tibial nails may incorporate options, based on fracture pattern and the needs of the surgeon, to avoid high-risk zones of the IPSN.
